# Annealing effects on the optical and morphological properties of ZnO nanorods on AZO substrate by using aqueous solution method at low temperature

**DOI:** 10.1186/1556-276X-9-632

**Published:** 2014-11-25

**Authors:** Da-Ren Hang, Sk Emdadul Islam, Krishna Hari Sharma, Shiao-Wei Kuo, Cheng-Zu Zhang, Jun-Jie Wang

**Affiliations:** 1 Department of Materials and Optoelectronic Science, National Sun Yat-sen University, 70 Lienhai Rd., Kaohsiung 804, Taiwan; 2 Center for Nanoscience and Nanotechnology, National Sun Yat-sen University, 70 Lienhai Rd., Kaohsiung 804, Taiwan

**Keywords:** Zinc oxide, Photoluminescence, Raman, Annealing

## Abstract

Vertically aligned ZnO nanorods (NRs) on aluminum-doped zinc oxide (AZO) substrates were fabricated by a single-step aqueous solution method at low temperature. In order to optimize optical quality, the effects of annealing on optical and structural properties were investigated by scanning electron microscopy, X-ray diffraction, photoluminescence (PL), and Raman spectroscopy. We found that the annealing temperature strongly affects both the near-band-edge (NBE) and visible (defect-related) emissions. The best characteristics have been obtained by employing annealing at 400°C in air for 2 h, bringing about a sharp and intense NBE emission. The defect-related recombinations were also suppressed effectively. However, the enhancement decreases with higher annealing temperature and prolonged annealing. PL study indicates that the NBE emission is dominated by radiative recombination associated with hydrogen donors. Thus, the enhancement of NBE is due to the activation of radiative recombinations associated with hydrogen donors. On the other hand, the reduction of visible emission is mainly attributed to the annihilation of OH groups. Our results provide insight to comprehend annealing effects and an effective way to improve optical properties of low-temperature-grown ZnO NRs for future facile device applications.

## Background

ZnO is a promising II-VI compound semiconductor because of its excellent catalytic, optoelectronic, and piezoelectric properties. It has been demonstrated to have diverse applications in electronic, optoelectronic, and electrochemical devices, such as ultraviolet (UV) lasers, light-emitting diodes, high-performance nanosensors, and solar cells [1-6]. In addition to the low cost, ease of availability, and chemical stability, the wide direct bandgap of 3.37 eV and large excitonic binding energy (60 meV at 300 K) make ZnO a highly competitive material to GaN. It was also reported that textured ZnO films may have higher quantum efficiency than GaN films [7]. Nowadays, ZnO thin films and nanostructures can be synthesized by using various deposition techniques, such as molecular beam epitaxy (MBE) [8], pulsed laser deposition (PLD) [9], metal-organic chemical vapor deposition (MOCVD) [10], chemical vapor deposition (CVD) [11-13], and aqueous solution deposition [14,15].

High-temperature techniques such as CVD and thermal evaporation have been mainly employed to grow aligned ZnO nanostructures, for example, nanorods (NRs). These processes have disadvantages of high energy consumption and requirement of expensive infrastructure. Here, we adopt an inexpensive and simple method to prepare uniformly distributed and well-aligned vertical ZnO NRs, whereas no catalyst or seeding step is required to initiate controlled growth. This approach is based on a one-step electrochemical processing of reliably nontoxic and abundant materials in aqueous solution at low temperature (≤80°C). Moreover, it allows for large-scale processing at low cost and facile integration for complex devices. The substrate of our choice is aluminum-doped zinc oxide (AZO). Meanwhile, transparent and conductive AZO substrate is an alternative to the ITO glass. AZO has better lattice matching than ZnO, so the development of ZnO/AZO devices is now a hot pursuit.

It is known that due to oxygen vacancies (V_O_), inherent n-type ZnO is formed and its carrier concentration depends on post-growth annealing treatment. The intrinsic defects in ZnO are always associated with various deposition processes. The understanding of defect properties is very useful to improve the quality of ZnO. Generally, post-deposition annealing treatment is a convenient and appropriate way to modify intrinsic defects and improve the crystallinity of ZnO. Proper annealing is an effective way to obtain high-quality ZnO material. There are many reports on the thermal treatment of ZnO for different annealing conditions such as annealing temperatures and gas environments to improve the optical properties of ZnO. In this paper, ZnO NRs were synthesized by an aqueous solution deposition method and effects of post-growth annealing were studied. The structural, morphological, and optical characteristics have been studied after annealing processes. Mechanisms that are responsible for the annealing effects are investigated.

## Methods

ZnO NRs were deposited on AZO substrates by an aqueous solution method. Zinc nitrate hexahydrate (Alfa Aesar, Ward Hill, MA, USA) was used as the zinc source. Ethanol amine (Merck, Whitehouse Station, NJ, USA) and hexamethylenetetramine (HMTA) were used as the stabilizer and base, respectively. Firstly, the AZO substrates were cleaned through sonication in a mixture of acetone and isopropyl alcohol (1:1), followed by cleaning with deionized water and drying in N_2_ atmosphere before use. The zinc precursor solution was prepared by dissolving equimolar zinc nitrate hexahydrate, HMTA, and ethanol amine in deionized water. The above solution was stirred by using a magnetic stirrer at 60°C for 10 min. NRs were grown by dipping the as-cleaned substrate horizontally into the prepared solution and were covered with a lid for 30 min at 80°C on a regular laboratory hot plate. The prepared NRs were annealed in air for 2 h by using a microprocessor-controlled furnace for different annealing temperatures ranging from 200°C to 600°C. X-ray diffraction (XRD) was conducted to examine the structure and orientation of ZnO. The surface morphology of the prepared NRs was investigated by scanning electron microscopy (SEM) (JEOL 6380, JEOL Ltd., Akishima-shi, Japan). For the photoluminescence (PL) investigations, the samples were excited by a chopped He-Cd laser beam working at 325 nm. The PL signal was dispersed by a Jobin Yvon Triax 550 monochromator (Jobin Yvon Inc., Edison, NJ, USA) equipped with a 2,400 rules/mm grating. A Hamamatsu R928 photomultiplier tube (Hamamatsu Photonics K.K., Iwata, Japan) equipped with a lock-in amplifier was used to record the optical intensity of the selected emission. A closed-cycle optical cryostat was used for low temperatures down to 10 K. Room-temperature (RT) Raman scattering measurements were performed in a backscattering configuration on a micro-Raman setup equipped with a Jobin Yvon iHR320 spectrometer and a multi-channel TE-cooled (−70°C) CCD detector.

## Results and discussion

### Evaluation of the as-grown sample

**Figure 1:**
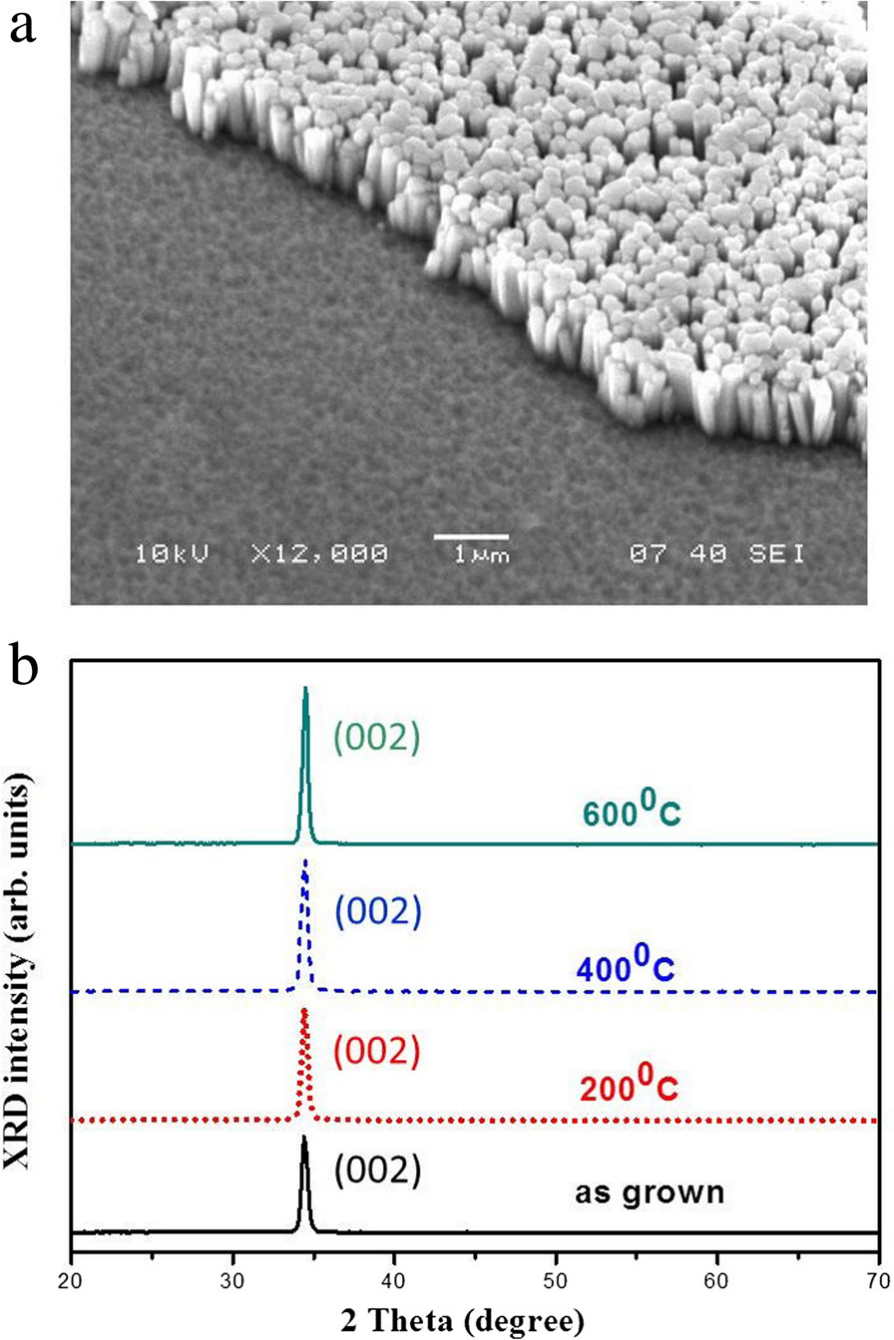
**SEM image and XRD patterns of the ZnO NRs on AZO. (a)** SEM image of the as-grown ZnO NRs on AZO. **(b)** XRD patterns of the as-grown and annealed ZnO NRs on AZO.

Figure 1a shows the tilt-view SEM image of the as-grown ZnO NRs on an AZO substrate. A high density of ZnO NRs grew vertically on the substrate. The diameter of the nanorods is about 200 nm. The crystallinity of the grown ZnO NRs was investigated by using XRD. As shown in Figure 1b, the XRD pattern of *θ*-2*θ* scan of the as-grown ZnO NRs shows only the ZnO (002) peak (black solid curve), indicating that the *c*-plane of ZnO is oriented parallel to the basal plane of the AZO substrate. It indicates that individual ZnO NRs, crystallized along the *c*-axis direction of ZnO, were all vertically aligned on the AZO substrate.

**Figure 2:**
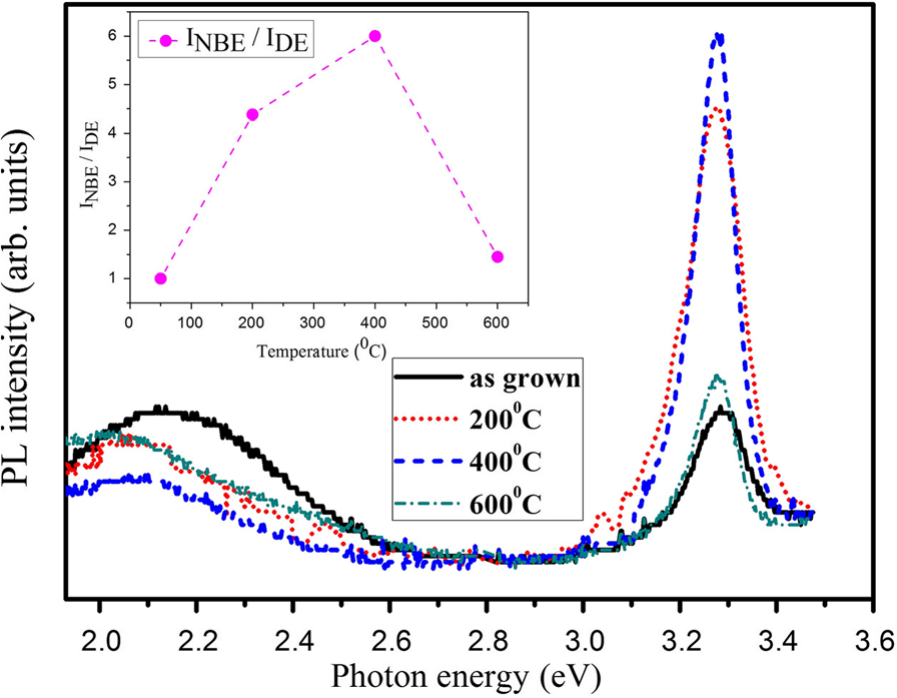
**The full spectra of RT PL of the as-grown and annealed ZnO NRs on AZO.** The inset shows the NBE to visible DE ratio against annealing temperature.

The black solid curve in Figure 2 shows the full RT PL spectrum of the as-prepared ZnO NRs. The PL spectrum comprises one UV emission band with a peak at 3.28 eV, which is attributed to the near-band-edge (NBE) emission. In addition, there is a broad visible emission band with comparable intensity, centered at approximately 2.15 eV, which can be ascribed to the defect emission (DE) [16,17]. It is frequently observed in ZnO prepared by aqueous solution and is often considered to be caused by atomic defects, such as oxygen vacancies in ZnO. It means that a lot of photo-generated carriers in ZnO NRs do not recombine close to the band edge, resulting in a poor NBE efficiency.

### Effects of annealing on structural and optical properties

**Figure 3:**
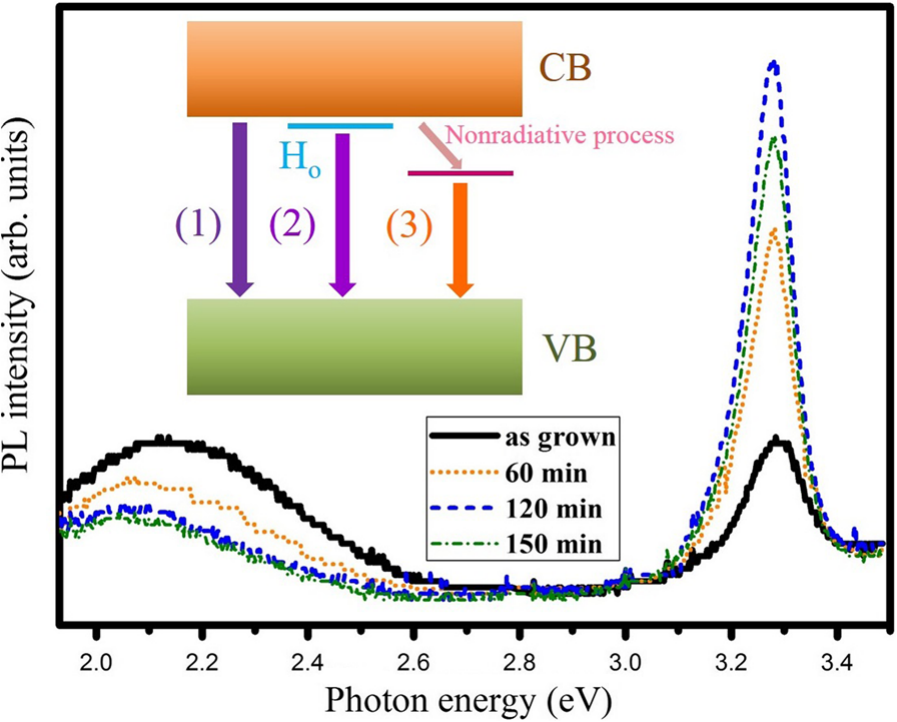
**RT PL spectra of ZnO NRs for different annealing duration at 400°C.** Inset: illustrative diagrams for recombination processes that have taken place in (1) NBE in the as-grown sample, (2) NBE in the annealed sample, and (3) defect emission accompanied by nonradiative recombination.

In order to improve the optical properties, we performed post-deposition thermal treatment in air at different temperatures for 2 h. Figure 1b displays XRD patterns of the annealed ZnO NRs. The intensities of the (002) diffractions are relatively higher for the samples annealed at various temperatures, suggesting the improvement of the crystalline quality. Then, the effects of thermal annealing on the PL properties of ZnO NRs were investigated. As Figure 2 shows, we found that there is pronounced influence on the NBE emission after annealing. The inset of Figure 2 shows the intensity ratio of NBE to visible DE against annealing temperature. It can be found that the ratio is 1, 4.38, 6, and 1.45 for the as-grown and 200°C-, 400°C-, and 600°C-annealed samples, respectively. We get the strongest NBE from the sample annealed at 400°C. However, the NBE decreases again with the high temperature treatment at 600°C. Moreover, it was noted that the annealing time of 2 h is the optimized duration. Figure 3 presents the RT PL spectra of the samples annealed at 400°C in different duration time ranging from 60 to 150 min. It shows that the intensity of NBE is enhanced with increasing annealing time until 120 min. However, it is weakened with prolonged annealing time.

**Figure 4:**
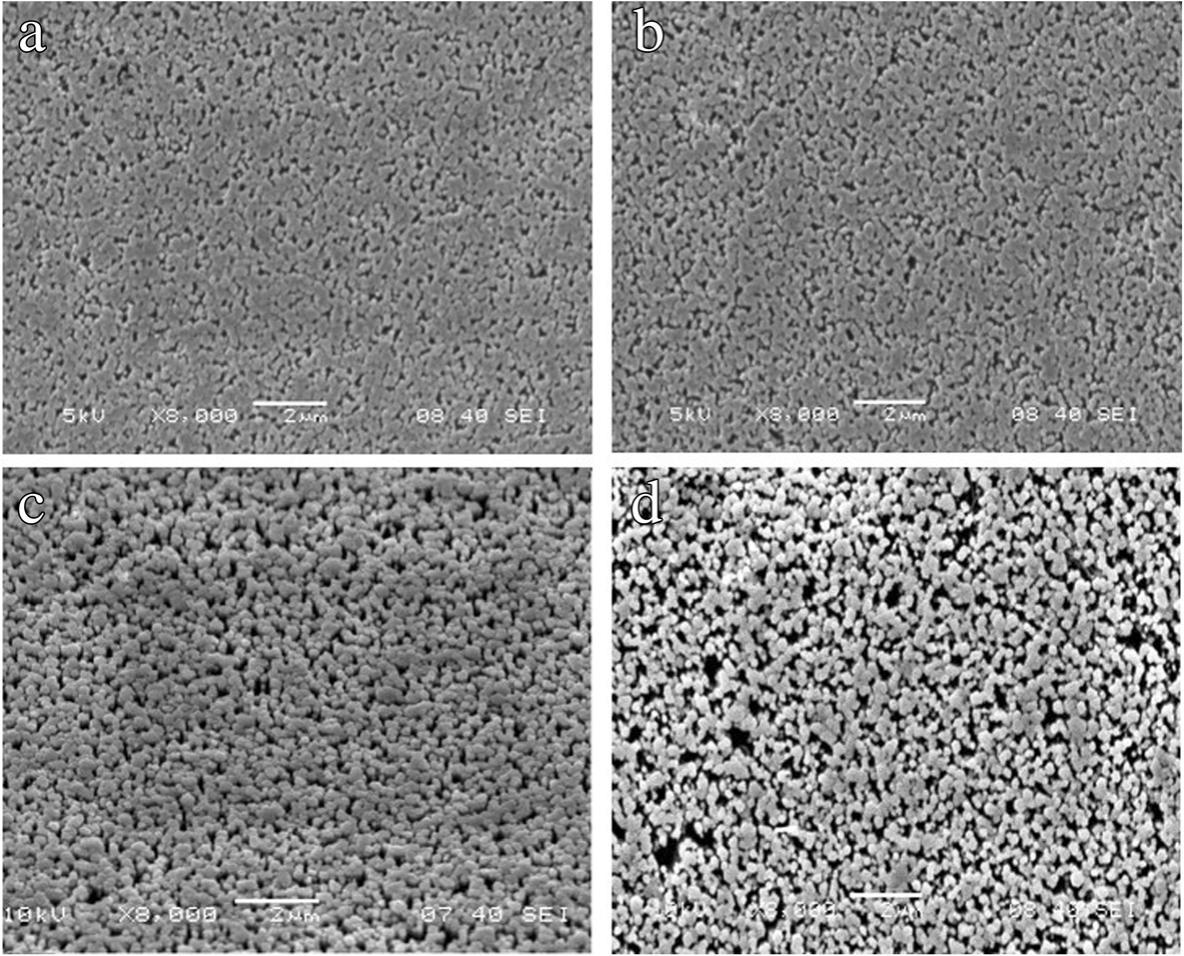
**SEM images. (a)** As-grown ZnO. Annealed ZnO in air at the following temperatures: **(b)** 200°C, **(c)** 400°C, and **(d)** 600°C.

To understand the influence of annealing on the morphological properties, we performed SEM measurements. Figure 4 shows the SEM images of the as-grown ZnO NRs and annealed ZnO NRs at various annealing temperatures. It can be observed that the hexagonal crystallite appears in all the samples, having average diameter ranging from 200 to 300 nm. The whole surface looks smooth and uniform in the nonannealed and annealed (at 200°C and 400°C) samples. However, at 600°C annealing temperature, surface smoothness and uniformity reduce dramatically and some void space is presented on the surface. It suggests that with increasing annealing temperatures, small crystallites start to coalesce together to form larger crystallites [18]. It may be attributed to the annealing-induced coalescence of small grains by grain boundary diffusion [18]. SEM images also indicate that hexagonal crystal phase is less distinct for the as-grown sample. Distinct hexagonal phase appears gradually with increasing annealing temperatures. Furthermore, it appears that the sample with 400°C annealing is best with respect to the homogeneous crystallinity. Together with previous PL results, it implies that 400°C is the optimum annealing temperature to get high-quality ZnO NRs.

### Low-temperature PL characterization

**Figure 5:**
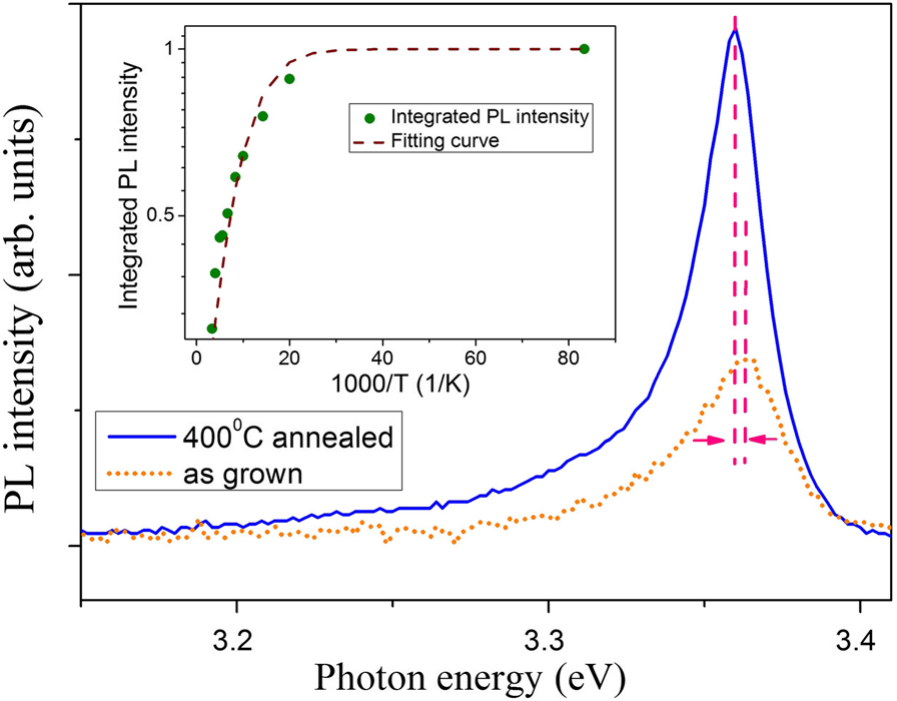
**The NBE spectra of the as-grown and annealed ZnO NRs on AZO at 10 K.** Inset: the integrated PL intensity of NBE as a function of reciprocal temperature for the annealed ZnO NRs, where the solid circles and the dashed line indicate experimental data and the fitting result to Equation 1, respectively.

**Figure 6:**
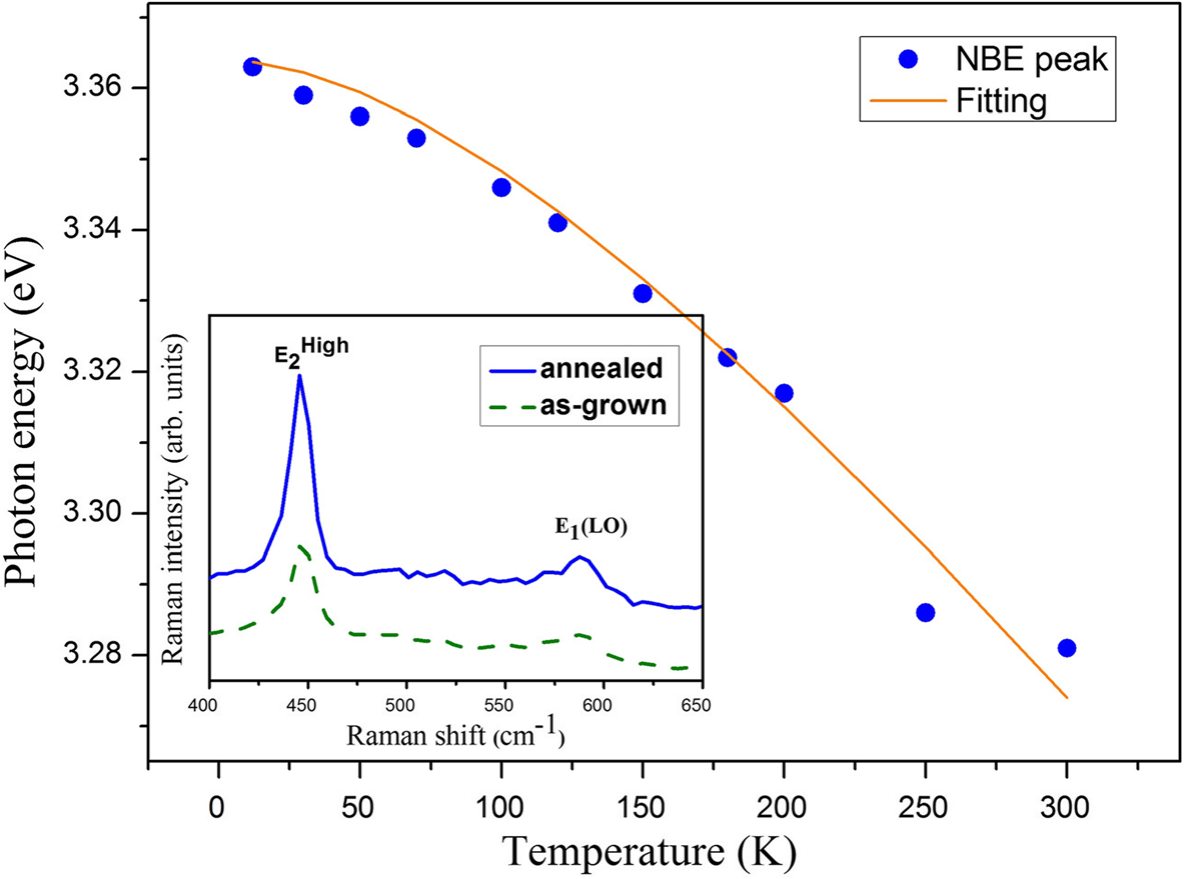
**The temperature dependence of NBE emission peak for the annealed ZnO NRs.** The solid line represents the fitting result to Equation 2. Inset: backscattering Raman spectra of the as-grown ZnO NRs and ZnO NRs after annealing at 400°C with scattering geometry *z*(*xx + xy*)*z*.

To reveal more optical properties, the 10 K PL spectra for the as-grown and 400°C-annealed samples are shown in Figure 5. It is clear that the NBE for the annealed sample is stronger and sharper than that for the as-grown sample. The full width at half maximum is 47 and 23.5 meV for the as-grown and 400°C-annealed samples, respectively. It gives a quantitative measure of the improved optical quality of our annealed sample. Moreover, we find that there are 3-meV redshifts of NBE emission after annealing at 400°C, as shown by the vertical dashed lines in Figure 5. In order to understand the nature of the enhanced NBE emission, we check the temperature-dependent activated behavior in the inset of Figure 5. In general, the emission efficiency is determined by the competition of radiative and nonradiative mechanisms. The radiative transition is assumed independent of temperature. On the other hand, the nonradiative contribution has a temperature dependence of exp(−*E*_a_/*k*_B_*T*), where *E*_a_ is the activation energy and *k*_B_ is the Boltzmann constant [19]. In this case, it leads to an expression described by the Arrhenius equation:

(1) $$I\left(T\right)=\frac{I_{0}}{1+P\exp\left(-E_{a}/k_{B}T\right)}\text{,}$$

where *I*(*T*) is the integrated PL intensity at *T* (K), *I*_0_ is a scaling factor, and *P* is a process rate parameter [19-21]. It provides important information to the origin of carrier recombination in various semiconductors [21-23]. The dashed line in the inset of Figure 5 is the least square fit of data with Equation 1. The fitted value of *P* and *E*_a_ is 5.2 and 19.5 meV, respectively. We obtain a high thermal activation energy. It is in close agreement with the activation energy of the hydrogen donor in ZnO deduced by PL (21 to 25 meV) [23,24]. Therefore, it is indicative that excitonic recombination at the hydrogen donor (H_O_) dominates the NBE after annealing treatment. Next, the temperature dependence of the emission peak *E*(*T*) was studied, as shown in Figure 6. It is known that the bandgap energy of ZnO decreases with increasing temperature. The change of bandgap energy with temperature is described by Varshni's empirical equation. Assuming that the peak positions of the NBE vary with the temperature as the energy bandgap, the dependence of *E*(*T*) on temperature can be fitted with the following expression:

(2) $$E\left(T\right)=E\left(0\right)-\frac{\alpha T^{2}}{\beta +T}\text{,}$$

where *E*(0) is the transition energy at zero temperature and *α* and *β* are fitting parameters referred to as Varshni's coefficients [20]. Both *α* and *β* are material-dependent. The *β* value is expected to be correlated with the Debye temperature, but a range of values were reported for ZnO [15,20,25]. The fitting results, which are denoted by the solid line in Figure 6, yield *E*(0) = 3.364 eV, *α* = 5.5 × 10^−4^ eV/K, and *β* = 250 K.

### Origin of enhanced optical properties

Based on the results above, we discuss annealing effects on the emission properties of our low-temperature-grown ZnO NRs. The enhancement of NBE by annealing might be explained by two previously proposed mechanisms. One is the elimination of unwanted functional groups acting as nonradiative centers on the surface of ZnO, and the other is the improvement of the crystal quality resulting from removal of intrinsic defects [26]. But the anomalous behavior after 400°C treatment, which shows reduced NBE, cannot be explained by the mechanisms above. In our aqueous solution growth of ZnO, generation of interstitial H is highly possible. Hydrogen defects can be introduced as a result of incomplete dehydration during the formation of ZnO [27,28]. Most of these initial hydrogen states, interstitial H or H complex (hydroxyl group and complex with other defects), are not active donors. During annealing at 400°C, intrinsic defects are passivated and initially trapped hydrogen is released. The interstitial H has high mobility and can move around the lattice. Since there are oxygen vacancies in the ZnO NRs, the interstitial hydrogen can be trapped inside oxygen vacancies, forming H_O_. Substitutional hydrogen at the oxygen site, from many experimental and theoretical investigations, is believed to be an important shallow donor in ZnO [23,28]. The existence of H_O_ exactly accounts for the observed activation energy in our PL study [23]. The decrease of NBE intensity under prolonged annealing or annealing higher than 400°C can then be attributed to the dissociation of hydrogen donors. Therefore, we attributed our NBE enhancement to the hydrogen donor formation activated by thermal annealing. The inset of Figure 3 shows illustrative diagrams for recombination processes that have taken place in (1) NBE in the as-grown sample and (2) NBE in the annealed sample. From the schematic diagrams, we can understand the redshift of NBE after annealing treatment, which is due to the annealing-induced activation of hydrogen donors.

The visible emission drops exactly in the opposite way as NBE enhances with annealing temperature, that is, it decreases with increasing annealing temperature up to 400°C and then it again increases at 600°C. Figure 3 also shows that visible emission decreases with increasing annealing time as well, but it is noted that after 120 min, further reduction of visible emission is insignificant. The chemical origin of the DE is intriguing. Our result shows that the broad DE is already reduced even with a low-temperature annealing at 200°C. It is indicative of the presence of OH groups whose desorption temperature is at approximately 150°C [29]. The assignment of this visible emission to the presence of hydroxyl groups is in agreement with a previous report on visible luminescence in ZnO nanocrystals [30]. Moreover, a previous study on the O-H local vibrational modes in ZnO also confirmed that such a low-temperature annealing results in the removal of OH groups [31]. Therefore, we attribute the corresponding reduction of visible (defect-related) emission to the annihilation of OH groups.

Finally, we carried out RT Raman scattering measurements to understand the light-scattering properties. Raman spectra for the nonannealed and 400°C-annealed samples are shown in the inset of Figure 6. Two dominant peaks have been clearly resolved. One peak is observed around 446 cm^−1^, which is attributed to the nonpolar optical phonon E_2_ (high) mode of wurtzite ZnO [21], and the other peak at 588 cm^−1^, known as E_1_ (LO), which is ascribed to the defect formation of oxygen vacancies [32-34]. No other mode related to defect-induced local vibration mode is observed. The weak intensity of E_1_ (LO) suggests that there are relatively low oxygen vacancies. Since both spectra contain E_1_ (LO) contribution, the low oxygen vacancies should be responsible for the residue visible emission after annealing treatment.

## Conclusions

In conclusion, we present the investigation of annealing effect on the optical and structural properties of vertically aligned ZnO NRs on AZO substrates by a single-step aqueous solution method at low temperature. The annealing temperature strongly affects both the NBE and visible emissions. We found the optimum annealing temperature to be 400°C. It yields a sharp and intense NBE emission and effectively suppressed visible emission. The enhancement of NBE is due to the activation of radiative recombinations associated with hydrogen donors while the reduction of DE is ascribed to the annihilation of OH groups. These results are useful to understand and optimize ZnO NRs grown in a low-temperature solution. Our approach has the advantages of low cost, fine quality, and straightforward one-step synthesis, which is promising to realize facile and controlled ZnO-based nanodevice fabrication.

## Competing interests

The authors declare that they have no competing interests.

## Authors’ contributions

DRH directed the project and finalized this manuscript. SEI carried out the sample preparation and XRD, SEM, PL, and Raman measurements. KHS and JJW helped perform the PL measurements. SWK provided equipment support in the synthesis work. CZZ helped prepare the samples. All the authors read and agreed with the final version of the paper.

## References

[B1] Alivov YI Özgür Ü Dogan S Johnstone D Avrutin V Onojima N Liu C Xie J Fan Q Morkoç H Photoresponse of *n-*ZnO/*p*-SiC heterojunction diodes grown by plasma-assisted molecular-beam epitaxy Appl Phys Lett 2005 86 241108 10.1063/1.1949730

[B2] Zhu H Shan CX Yao B Li BH Zhang JY Zhao DX Shen DZ Fan XW High spectrum selectivity ultraviolet photodetector fabricated from an n-ZnO/p-GaN heterojunction J Phys Chem C 2008 112 20546 10.1021/jp808870z

[B3] Hsueh HT Chang SJ Weng WY Hsu CL Hsueh TJ Hung FY Wu SL Dai BT Fabrication and characterization of coaxial p-copper oxide/n-ZnO nanowire photodiodes IEEE Trans Nanotechnol 2012 11 127

[B4] Huang H Fang G Mo X Yuan L Zhou H Wang M Xiao H Zhao X Zero-biased near-ultraviolet and visible photodetector based on ZnO nanorods/*n*-Si heterojunction Appl Phys Lett 2009 94 063512 10.1063/1.3082096

[B5] Chu S Lim JH Mandalapu LJ Yang Z Li JL Sb-doped p-ZnO/Ga-doped n-ZnO homojunction ultraviolet light emitting diodes Appl Phys Lett 2008 92 152103 10.1063/1.2908968

[B6] Wu J-K Chen W-J Chang Y-H Chen Y-F Hang D-R Liang C-T Lu J-Y Fabrication and photo-response of ZnO nanowiress/CuO coaxial heterojunction Nanoscale Res Lett 2013 8 387 10.1186/1556-276X-8-387 24044381 PMC3850089

[B7] Yu P Tang ZK Wong GK Segawa Y Kawasaki M Scheffler M, Zimmermann R Stimulated emission at room temperature from ZnO quantum dot films ICPS'96. 23th International Conference on the Physics of Semiconductors: 21-26 July 1996; Berlin 1996 Singapore: World Scientific 1453

[B8] Wang H-C Liao C-H Chueh Y-L Lai C-C Chen L-H Tsiang RC-C Synthesis and characterization of ZnO/ZnMgO multiple quantum wells by molecular beam epitaxy Opt Mater Express 2013 3 237 10.1364/OME.3.000237

[B9] Gluba MA Nickel NH Hinrichs K Rappich J Improved passivation of the ZnO/Si interface by pulsed laser deposition J Appl Phys 2013 113 043502 10.1063/1.4788675

[B10] Hauschild R Lange H Priller H Klingshirn C Kling R Wang A Fan HJ Zacharias M Kalt H Stimulated emission from ZnO nanorods Phy Status Solidi B 2006 243 853 10.1002/pssb.200564718

[B11] Chou MM-C Hang D-R Chen C Wang SC Lee CY Nonpolar a-plane ZnO growth and nucleation mechanism on (1 0 0) (La, Sr)(Al, Ta)O_3_ substrate Mater Chem Phys 2011 125 791 10.1016/j.matchemphys.2010.09.057

[B12] Chou MM-C Hang D-R Chen C Liao YH Epitaxial growth of nonpolar m-plane ZnO (10–10) on large-size LiGaO_2_ (100) substrates Thin Solid Films 2010 519 3627

[B13] Chen C Lan YT Chou MM-C Hang D-R Yan T Feng H Lee CY Chang SY Li CA Growth and characterization of vertically aligned nonpolar [11̅00] orientation ZnO nanostructures on (100) γ-LiAlO_2_ substrate Cryst Growth Des 2012 12 6208 10.1021/cg301394x

[B14] Ting C-C Li C-H Kuo C-Y Hsu C-C Wang H-C Yang M-H Compact and vertically-aligned ZnO nanorod thin films by the low-temperature solution method Thin Solid Films 2010 518 4156 10.1016/j.tsf.2009.11.082

[B15] Chen W-J Wu J-K Lin J-C Lo S-T Lin H-D Hang D-R Shih MF Liang C-T Chang YH Room-temperature violet luminescence and ultraviolet photodetection of Sb-doped ZnO/Al-doped ZnO homojunction array Nanoscale Res Lett 2013 8 313 10.1186/1556-276X-8-313 23826909 PMC3711895

[B16] Alvi NUH Hussain S Jensen J Nur O Willander M Influence of helium-ion bombardment on the optical properties of ZnO nanorods/p-GaN light-emitting diodes Nanoscale Res Lett 2011 6 628 10.1186/1556-276X-6-628 22152066 PMC3274553

[B17] Özgür Ü Alivov YI Liu C Teke A Reshchikov MA Doğan S Avrutin V Cho SJ Morkoç H A comprehensive review of ZnO materials and devices J Appl Phys 2005 98 041301 10.1063/1.1992666

[B18] Sengupta J Sahoo RK Mukherjee CD Effect of annealing on the structural, topographical and optical properties of sol–gel derived ZnO and AZO thin films Mater Lett 2012 83 84

[B19] Pankove JI Optical Properties in Semiconductors 1971 New York: Dover

[B20] Hang D-R Islam SE Sharma KH Chen C Liang C-T Chou MM-C Optical characteristics of nonpolar *a*-plane ZnO thin film on (010) LiGaO_2_ substrate Semicond Sci Tech 2014 29 085004 10.1088/0268-1242/29/8/085004

[B21] Hang D-R Sharma KH Islam SE Chen C Chou MM-C Resonant Raman scattering and photoluminescent properties of nonpolar *a*-plane ZnO thin film on LiGaO_2_ substrate Appl Phys Express 2014 7 041101 10.7567/APEX.7.041101

[B22] Hang D-R Chou MM-C Chang L Lin JL Heuken M Optical characteristics of *m*-plane InGaN/GaN multiple quantum well grown on LiAlO_2_ (100) by MOVPE J Cryst Growth 2009 311 2919 10.1016/j.jcrysgro.2009.01.042

[B23] Huang XH Tay CB Zhan ZY Zhang C Zheng LX Venkatesan T Chua SJ Universal photoluminescence evolution of solution-grown ZnO nanorods with annealing: important role of hydrogen donor Cryst Eng Comm 2011 13 7032 10.1039/c1ce05882g

[B24] Dev A Richters JP Sartor J Kalt H Gutowski J Voss T Enhancement of the near-band-edge photoluminescence of ZnO nanowires: important role of hydrogen incorporation versus plasmon resonances Appl Phys Lett 2011 98 131111 10.1063/1.3569951

[B25] Lee SH Lee JS Ko WB Sohn JI Cha SN Kim JM Park YJ Hong JP Photoluminescence analysis of energy level on Li-doped ZnO nanowires grown by a hydrothermal method Appl Phys Express 2012 5 095002 10.1143/APEX.5.095002

[B26] Yang LL Zhao QX Willander M Yang JH Ivanov I Annealing effects on optical properties of low temperature grown ZnO nanorod arrays J Appl Phys 2009 105 053503 10.1063/1.3073993

[B27] Brauer G Anwand W Grambole D Skorupa W Hou Y Andreev A Teichert C Tam KH Djurisic AB Non-destructive characterization of vertical ZnO nanowire arrays by slow positron implantation spectroscopy, atomic force microscopy, and nuclear reaction analysis Nanotechnology 2007 18 195301 10.1088/0957-4484/18/19/195301

[B28] Du MH Biswas K Anionic and hidden hydrogen in ZnO Phys Rev Lett 2011 106 115502 21469877 10.1103/PhysRevLett.106.115502

[B29] Xie R Sekiguchi T Ishigaki T Ohashi N Li D Yang D Liu B Bando Y Enhancement and patterning of ultraviolet emission in ZnO with an electron beam Appl Phys Lett 2006 88 134103 10.1063/1.2189200

[B30] Norberg NS Gamelin DR Influence of surface modification on the luminescence of colloidal ZnO nanocrystals J Phys Chem B 2005 109 20810 10.1021/jp0535285 16853697

[B31] Shi GA Stavola M Pearton SJ Thieme M Lavrov EV Weber J Hydrogen local modes and shallow donors in ZnO Phys Rev B 2005 72 195211

[B32] Exarhos GJ Sharma SK Influence of processing variables on the structure and properties of ZnO films Thin Solid Films 1995 270 27 10.1016/0040-6090(95)06855-4

[B33] Xing YJ Xi ZH Xue ZQ Zhang XD Song JH Wang RM Xu J Song Y Zhang SL Yu DP Optical properties of the ZnO nanotubes synthesized via vapor phase growth Appl Phys Lett 2003 83 1689 10.1063/1.1605808

[B34] Rajalakshmi M Arora AK Bendre BS Mahamuni S Optical phonon confinement in zinc oxide nanoparticles J Appl Phys 2000 87 2445 10.1063/1.372199

